# Triggerfish Recording of IOP Patterns in Combined HFDS Minimally Invasive Glaucoma and Cataract Surgery: A Prospective Study

**DOI:** 10.3390/jcm10163472

**Published:** 2021-08-06

**Authors:** Bojan Pajic, Mirko Resan, Brigitte Pajic-Eggspuehler, Horace Massa, Zeljka Cvejic

**Affiliations:** 1 Eye Clinic Orasis, Swiss Eye Research Foundation, 5734 Reinach AG, Switzerland; brigitte.pajic@orasis.ch; 2 Department of Physics, Faculty of Sciences, University of Novi Sad, Trg Dositeja Obradovica 4, 21000 Novi Sad, Serbia; zeljka.cvejic@df.uns.ac.rs; 3 Department of Clinical Neurosciences, Division of Ophthalmology, Geneva University Hospitals, 1205 Geneva, Switzerland; horace.massa@hcuge.ch; 4 Faculty of Medicine, University of Geneva, 1205 Geneva, Switzerland; 5 Faculty of Medicine of the Military Medical Academy, University of Defense, 11000 Belgrade, Serbia; resan.mirko@gmail.com

**Keywords:** Triggerfish, IOP pattern, minimal invasive glaucoma surgery (MIGS), high frequency deep sclerotomy (HFDS)

## Abstract

Background: The aim of the study is to investigate whether the circadian IOP rhythm can be influenced by combined cataract surgery with high frequency deep sclerotomy (HFDS) and whether intraocular pressure (IOP) can be significantly reduced by HFDS. Methods: In our study 10 patients were included, in whom 24 h IOP monitoring was installed before and after HFDS/cataract surgery using a Triggerfish. HFDS is a minimally invasive glaucoma surgery (MIGS). Results: After performed HFDS combined with cataract surgery, the IOP was reduced from 27.7 ± 2.11 mmHg to 14.4 ± 2.59 mmHg, which is highly significant (*p* < 0.001). The contact lens sensor (CLS) cosinor analysis pre- and postoperatively showed that the circadian rhythm is not influenced by the surgery, i.e., the circadian IOP rhythm did not show significant differences before and after surgery. Conclusions: HFDS combined with cataract surgery is a potent surgical method that can significantly reduce the IOP. However, the circadian rhythm cannot be changed by the surgery. The acrophase remained during the night in all patients.

## 1. Introduction

Open angle glaucoma is a multifactorial disease in which different risk factors are involved. There are several ways to treat open-angle glaucoma, the first of which is intraocular pressure (IOP) reduction where progression of the disease is significantly slowed or even kept stable [1,2]. There is strong evidence that high IOP is associated with incidence, prevalence, and progression of glaucoma [3,4,5,6,7]. Other important risk factors for glaucoma progression are ocular blood flow and the imbalance between IOP and blood pressure [8,9]. IOP peaks and fluctuations are considered to be other important risk factors [9]. For all these reasons, it is not only important to achieve a sufficient and significant reduction in IOP, but also to consider their behaviour in the circadian cycle.

A significant proportion of patients experience visual impairment due to glaucoma despite the IOP being in a good range during office hours. It was found that 69% of IOP peaks occur outside office hours [10]. A 24 h tonometry can give us much more information about the IOP behaviour of each individual patient in contrast to an IOP evaluation during consultation hours or hospitalisation, where the non-physiological behaviour of the patient is observed. Optimal would be a 24 h IOP measurement under physiological conditions independent of body position or state of wakefulness [11].

To overcome these limitations, a contact lens sensor (CLS, SENSIMED Triggerfish, Sensimed AG, Lausanne, Switzerland) was developed, whose measurement is based on the fact that the IOP is correlated with changes in ocular dimensions.

IOP follows a circadian rhythm, an internal clock that allows for alignment of biological process with a 24 h light-dark cycle [12]. Cataract and glaucoma are ophthalmic diseases that might affect photic input to the circadian system. These diseases lead to visual impairment and indirectly cause physical inactivity, which can further cause sleep problems and daytime sleepiness.

It can be shown that the circadian IOP course is like a fingerprint, even under treatment conditions of glaucoma. In our previous studies, not only could the individual circadian rhythm of the patients be shown, but also the nightly peaks [13]. In another study, a 24 h CLS measurement showed that the rate of visual field progression in previously treated glaucoma eyes could be correlated based on a specific time of treatment. It is possible that 24 h CLS measurement may be used to identify an important risk factor for glaucoma progression in order to initiate appropriate treatment [14]. In other words, 24 h CLS measurement has the potential to identify other risk factors that have a direct therapeutic impact on glaucoma treatment.

These collected data by biometric sensors are valuable to the extent that the understanding of a treatment method can be significantly improved. Now that we have a new measurement tool at our disposal, the question arises as to which treatments have an impact on the circadian IOP rhythm and which do not. This could lead to further refinement of glaucoma treatment strategies.

## 2. Materials and Methods

**Figure 1:**
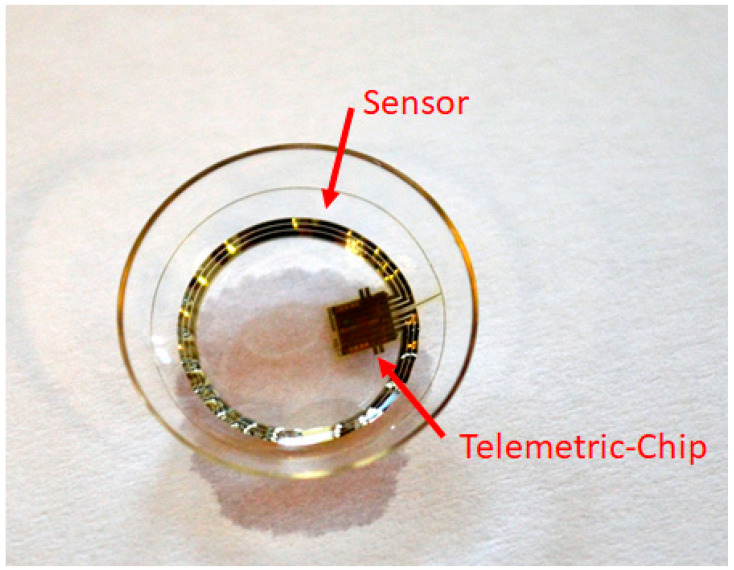
Triggerfish contact lens with sensor and Bluetooth.

In a prospective, open label study we assessed the IOP fluctuations as recorded with SENSIMED Triggerfish^®^ (Figure 1) in one eye for two 24 h periods at three month intervals before and after HFDS combined with phacoemulsification in patients with primary open angle glaucoma and cataract. The same eye was recorded on both occasions. Patients performed a baseline 24 h IOP monitoring within one week before and one to three months after surgery. A total of 10 patients were included in the study. The following inclusion criteria were defined for the conduct of the study: diagnosis of open angle glaucoma, documented glaucomatous visual field damage with MD of −3 dB or worse and/or findings at the optic nerve head and/or retinal nerve fiber layer that are representative of glaucoma, stable IOP-lowering treatment for the preceding 4 weeks, older than 18 years at inclusion, not more than six spherical diopters or equivalent and no more than two cylindrical diopters, no surgery within the last three months, and signed informed consent for the investigation. The following points were defined as exclusion criteria: corneal or conjunctival abnormality hindering contact lens adaptation, severe dry eye syndrome, patients allergic to silicone, participation in other clinical research within the last four weeks, advanced-stage glaucoma, patients with baseline IOP > 30 mmHg under treatment, Monokel situation, and pregnancy.

SENSIMED Triggerfish^®^ is a CE-marked device manufactured by Sensimed AG (Lausanne, Switzerland). The system consists of a telemetric sensor based on a soft silicone contact lens (CLS) that is placed on the patient’s eye, an antenna that is placed around the patient’s eye, and a data cable connected to an antenna and to the recorder that powers the device and stores the recorded data. The SENSIMED Triggerfish^®^ is intended as a tool for the clinician to continuously record the timing of relative changes believed to be related to IOP.

The sensing resistive gauges (i.e., strain gauges in a Wheatstone bridge configuration that measure changes in electric resistance/voltage) have a circular arc shape around the centre of CLS, placed at the position that corresponds to the cornea-sclera junction, where IOP changes are assumed to involve maximum corneal deformation (Figure 1).

Earlier studies have demonstrated that the Triggerfish CLS provided reproducible data [15,16]. Furthermore, several clinical studies have provided information about satisfactory tolerance of the Triggerfish CLS in both healthy and glaucomatous patients [17,18].

According to [19,20,21], 3 μm change in the radius of curvature of the cornea (over a typical radius of 7.8 mm) corresponds to 1 mmHg in IOP.

Measurements are recorded for 30 s every 5 min during a 24 h period, which leads to 288 measurements over a 24 h time period. This measuring method can be assumed to be pseudo-continuous, which, in contrast to standard static measurements performed with Goldmann tonometry, provides much more accurate data on the treatment, evolution, and surveillance of glaucoma. The measurement data is transmitted wirelessly from the CLS to an antenna that is taped to the head. From there, the information is transferred to a data storage device that is worn around the neck. After 24 h, the CLS is removed and the data is analysed with appropriate software.

**Figure 2:**
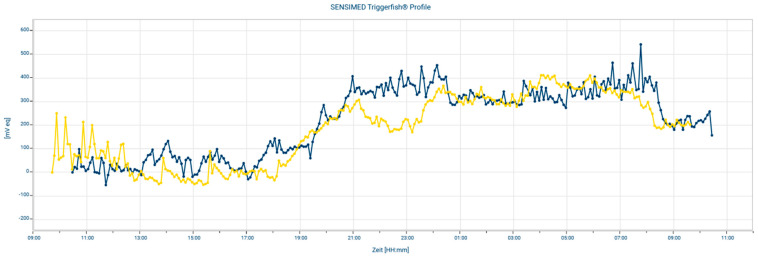
A measurement example overlays a 24 h measurement before and after the surgery.

Circadian rhythm measurement is based on cosinor-based rhythmometry, which is commonly used to study circadian biological rhythms. Using an example, a study patient, we showed the measurement of circadian biological rhythms before and after surgery (Figure 2). These measured graphs can be superimposed. It is obvious that the circadian biological rhythm in this example follows the same rules as before the surgery, regardless of the treatment. The CLS measurement was performed in the same way for all patients and all values were used for the evaluation.

A combined cataract/high frequency deep sclerotomy (HFDS) was performed in all patients. For the cataract surgery, an Oertli Catarhex 3 with an easy tip with a diameter of 2.2 mm was used. The Phaco-Chop was used as the surgical technique. At the end of the cataract surgery, HFDS was applied. Before inserting the HFDS tip through the paracentesis, the anterior chamber is filled using viscoelastic. Visual control in the operating area is ensured by means of four-mirror glass. With the HFDS tip, six sclerotomies of 0.3 mm thickness, 0.6 mm width, and a depth of 1 mm are formed.

**Figure 3:**
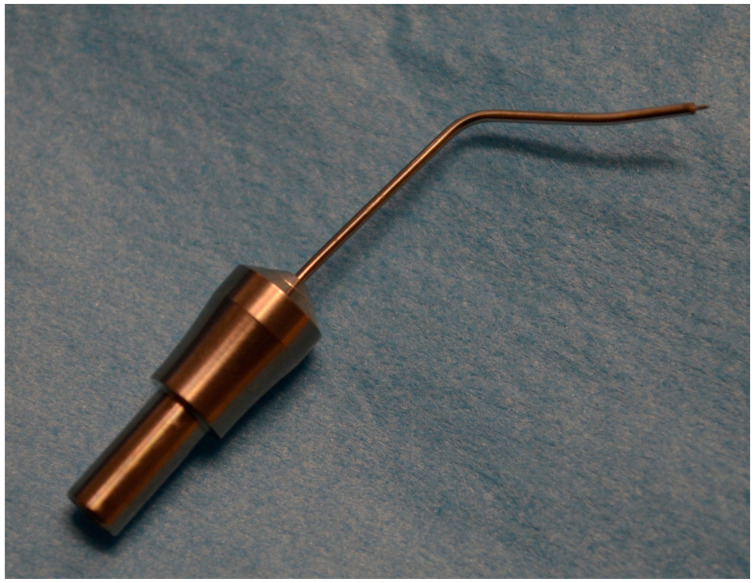
High Frequency Diathermy Probe (HFDS).

The high frequency diathermy probe (Figure 3) has an internal platinum electrode that is insulated from one of the returning coaxial electrodes. The platinum tip has a length of 1 mm and a width of 0.3 mm and is inclined at an angle of 15°. The outer diameter of the probe is 0.9 mm. The high frequency current flows at 500 KHz, which generates a temperature of 130 °C at the tip of the probe. A bipolar current generator forms an electric field only at the tip of the probe. Consequently, also due to the high frequency, the temperature propagation is only local around the probe tip. No coagulation effects could be detected in the surrounding area.

Corneal pachymetry (CCT) was measured in the study using the Scheimpflug camera Pentacam (Oculus Optikgeräte GmbH, Wetzlar, Germany). IOP was measured using Goldmann tonometry.

This study was performed in accordance with the protocols of the Declaration of Helsinki, internationally recognized standards for clinical research involving medical devices, and all applicable regulatory requirements. The study was approved by the institutional review board and ethics committee of the Faculty of Medicine of the Military Medical Academy, University of Defense, Belgrade, Serbia.

Statistical analysis was performed using IBM SPSS Statistics version 22.0 (IBM Corp., Armonk, NY, USA). The Kolmogorov–Smirnov test and the Shapiro–Wilk test were used to determine whether there is a normal distribution of the data (a data set is normally distributed if *p* > 0.05). Parametric datasets are further analysed with the Student-*t* test and non-parametric datasets with the Wilcoxon test. Significance is given if *p* < 0.05.

## 3. Results

The study included 10 patients with open angle glaucoma, 5 subjects (50%) were female and 5 subjects (50%) were male. The mean age of the patients was 68.3 ± 6.43 (range 55–81) years. The preoperative cornea pachymetry CCT could be determined with a mean value of 557 ± 13.5 µm (range 540–570 µm) (Table 1).

**Figure 4:**
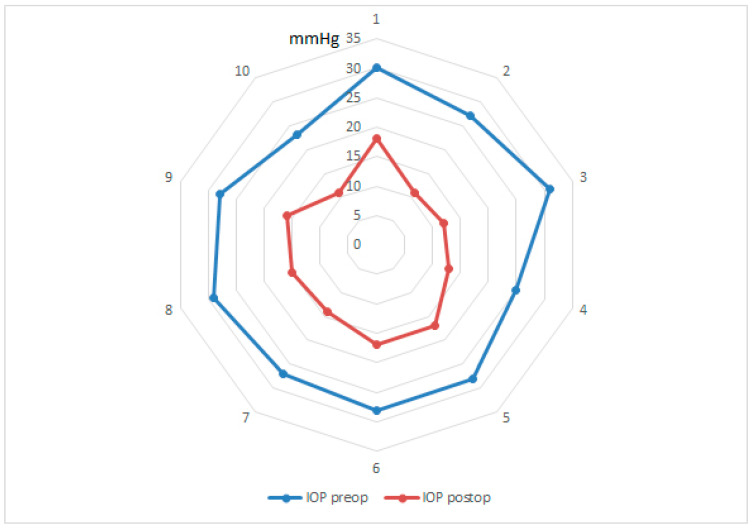
Star diagram with IOP values in mmHg before and after the operation for each subject.

The preoperative IOP averaged 27.7 ± 2.11 mmHg (range 24–30 mmHg). After combined cataract surgery and HFDS, the IOP was reduced to 14.4 ± 2.59 mmHg (range 11–18 mmHg) three months postoperatively, which is significant (*p* < 0.001). Preoperatively, an average of 3.1 ± 0.99 ocular pressure reducing drugs had to be administered. After three months, no patient needed an ocular pressure-lowering treatment, which is highly significant (*p* < 0.001). This corresponds to an average IOP reduction of 13.3 mmHg or a reduction of 48%. A significant IOP reduction was achieved in all patients (Figure 4).

The CLS cosinor analysis pre- and postoperatively of the maximum amplitude of the acrophase in all patients in the sleeping period phase was achieved and the minimum amplitude of the bathyphase in the afternoon was determined. The acrophase was 155.6 ± 76.47 mVeq preoperatively and 145.7 ± 59.17 mVeq postoperatively, which is not statistically significant (*p* = 0.66). On average, the acrophase was detected preoperatively at 03 h 24 min, whereas postoperatively it was slightly earlier at 02 h 32 min. The same phenomenon could also be observed in the bathyphase where the amplitude was on average preoperatively at 15 h 32 min and shifted to earlier at 14 h 17 min. The CLS analysis of the biphasic amplitude of the acrophase had preoperative mean of 166.6 ± 74.93 mVeq and postoperative mean of 172.8 ± 62.53 mVeq which was also not significant (*p* = 0.79).

**Figure 5:**
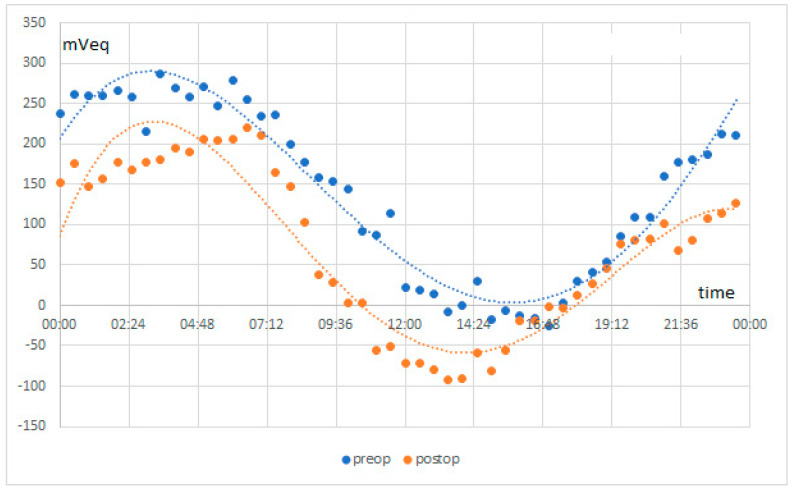
Triggerfish measurement results in mVeq of all patients before and after surgery.

The analysis of the amplitudes at any time of the 24 h measurement pre- and postoperatively have been performed and compared. It could be shown that there was no significant statistical difference at any time (*p* > 0.05). In other words, it can be concluded that the surgical treatment by cataract surgery combined with HFDS does not change the circadian rhythm (Figure 5).

## 4. Discussion

We found that after cataract surgery combined with HFDS, a minimally invasive glaucoma surgery, we achieved an excellent pressure reduction of 13.3 mmHg. In comparison, it has been described several times in the literature that cataract surgery itself causes an IOP reduction of 3 mmHg in open angle glaucoma [22,23]. Consequently, HFDS leads to an outstanding IOP reduction. Triggerfish measurement data are given in mVeq and are associated with IOP fluctuation. We found that cataract surgery combined with HFDS does not alter the circadian rhythm, although it can be said that pre- and postoperative circadian rhythms statistically non-significantly differ. Interestingly, however, the amplitude maximum of the acrophase in the night postoperative could be detected 52 min earlier compared to the preoperative data. Accordingly, the bathyphase in the afternoon was shifted to 75 min earlier preoperatively versus postoperatively. This result makes sense, since cataract surgery combined with HFDS significantly reduces the outflow resistance without affecting the circadian rhythm. It is important to know that HFDS does not change the circadian IOP cycle. Therefore, the postoperative target eye pressure should ideally always be lower than the IOP peak. This has a direct impact on the diagnosis and has therapeutic consequences.

Remarkably similar results could be shown after selective laser trabeculoplasty (SLT) in open angle glaucoma, when the absolute IOP value was reduced by the treatment but the circadian rhythm remained unchanged [24]. In another study, it was described that surgical treatment of open angle glaucoma showed a smaller IOP fluctuation postoperatively than following conservative drug treatment. However, the surgical group was inhomogeneous in the sense that different surgical methods were used such as deep sclerectomy, trabeculectomy, and ex-press (shunt) [25]. We cannot confirm this because the preoperative amplitudes of the circadian rhythm in our study do not differ significantly after surgery. This was also shown in another study, which states that the potential for surgical IOP reduction by deep sclerectomy and trabeculectomy is greater than the use of drug treatment with Latanoprost, but that the treatment itself does not cause a difference in IOP fluctuation [26]. In principle, however, it would be desirable that the treatment have an influence on the circadian rhythm in the sense of a smaller postoperative IOP fluctuation, which would potentially cause less damage to the optic nerve. Basically, it should be mentioned that a typical pattern of the contact lens sensor (CLS) with a nocturnal rise in IOP also exists in healthy patients [27,28]. Furthermore, in another paper it was concluded that IOP measurement several times a day does not sufficiently reflect its dynamics, which clearly shows the advantage of CLS measurement [29]. The 24 h measurement by CLS is very useful to determine the nocturnal associated IOP peak and consequently to improve the clinical management of glaucoma therapeutically, especially in patients showing progression [30]. Additionally, the characteristic that both eyes behave symmetrically in the sense that the IOP fluctuations, peak and amplitudes, have the same values is important [31].

An important aspect to discuss is how reliable the received data from CLS is. In a very good study design, a CLS measurement was installed in one eye while the IOP measurement was performed on the opposite eye with a pneumatonometer during a 24 h period [32]. The CLS uses a strain gauge, which is installed in a contact lens. The circumferential curvature of the corneoscleral part of the bulbus is measured [21,33]. At present, there is no complete understanding between an IOP change and the volume change measured in the corneoscleral region. However, there seems to be a correlation [32]. The pneumatonometer effectively measures the IOP by applanation of the central cornea through externally applied pressure, where the back pressure can be measured as IOP [34]. The pneumatonometer was applied every 2 h. The cosinor analysis of the nocturnal peak time of the 24 h measurement by CLS and the calculated analysis of the IOP measured in the contralateral eye using a pneumatonometer did not differ significantly [22]. In this sense, the measured amplitude profiles in our study before and after surgery can be considered reliable. Our measured values are reproducible, which is a great advantage in terms of the value of the treatment carried out.

An advantage of our study is that it was conducted prospectively. However, it should be noted that the number of eligible patients who participated in this study was only ten, which is a limitation of our research. Nevertheless, our results show high validity. Regarding IOP, it seems clear that it decreased after the surgical treatment described above, which did not affect the change in circadian rhythm.

## 5. Conclusions

In our study, we found that cataract surgery combined with high frequency deep sclerotomy (HFDS) achieved a highly significant IOP reduction. However, it was found that the circadian rhythm measured by CLS is not changed by surgery compared to preoperative analysis. It has also been observed that all patients in both cases, before and after surgery, had the highest IOP during the nocturnal period.

## Figures and Tables

**Table 1:** Preoperative baseline values.

Sex	Age	Pachymetry CCT	IOP Preoperative	IOP Reducing Drugs Preoperative
5 female, 5 male	68.3 ± 6.43 years (range 55–81)	557 ± 13.5 µm (range 540–570)	27.7 ± 2.11 mmHg (range 24–31)	3.1 ± 0.99

## Data Availability

The data presented in this study are available on request from the authors, in particular the datasets are archived in the clinics treated. The data are not publicly available as they contain information that could compromise the privacy of the participants.

## References

[1] Peters D., Bengtsson B., Heijl A. (2014). Factors associated with lifetime risk of open-angle glaucoma blindness. Acta Ophthalmol..

[2] (2017). European Glaucoma Society Terminology and Guidelines for Glaucoma, 4th Edition—Chapter 2: Classification and terminology Supported by the EGS Foundation: Part 1: Foreword; Introduction; Glossary; Chapter 2 Classification and Terminology. Br. J. Ophthalmol..

[3] Gordon M.O., Beiser J.A., Brandt J.D., Heuer D.K., Higginbotham E.J., Johnson C.A., Keltner J.L., Miller J.P., Parrish R.K., Wilson M.R. (2002). The Ocular Hypertension Treatment Study: Baseline factors that predict the onset of primary open-angle glaucoma. Arch. Ophthalmol..

[4] Miglior S., Pfeiffer N., Torri V., Zeyen T., Cunha-Vaz J., Adamsons I. (2007). Predictive factors for open-angle glaucoma among patients with ocular hypertension in the European Glaucoma Prevention Study. Ophthalmology.

[5] Leske M.C., Heijl A., Hyman L., Bengtsson B., Dong L., Yang Z., EMGT Group (2007). Predictors of long-term progression in the early manifest glaucoma trial. Ophthalmology.

[6] De Moraes C.G., Juthani V.J., Liebmann J.M., Teng C.C., Tello C., Susanna R., Ritch R. (2011). Risk factors for visual field progression in treated glaucoma. Arch. Ophthalmol..

[7] Costa V.P., Harris A., Anderson D., Stodtmeister R., Cremasco F., Kergoat H., Lovasik J., Stalmans I., Zeitz O., Lanzl I. (2014). Ocular perfusion pressure in glaucoma. Acta Ophthalmol..

[8] Kurvinen L., Kytö J.P., Summanen P., Vesti E., Harju M. (2014). Change in retinal blood flow and retinal arterial diameter after intraocular pressure reduction in glaucomatous eyes. Acta Ophthalmol..

[9] Asrani S., Zeimer R., Wilensky J., Gieser D., Vitale S., Lindenmuth K. (2000). Large diurnal fluctuations in intraocular pressure are an independent risk factor in patients with glaucoma. J. Glaucoma.

[10] Barkana Y., Anis S., Liebmann J., Tello C., Ritch R. (2006). Clinical utility of intraocular pressure monitoring outside of normal office hours in patients with glaucoma. Arch. Ophthalmol..

[11] Prata T.S., De Moraes C.G., Kanadani F.N., Ritch R., Paranhos A. (2010). Posture-induced intraocular pressure changes: Considerations regarding body position in glaucoma patients. Surv. Ophthalmol..

[12] Lam V., Chin J. (2017). Evolution and Design of Invertebrate Circadian Clock.

[13] Pajic B., Pajic-Eggspuehler B., Haefliger I. (2011). Continuous IOP fluctuation recording in normal tension glaucoma patients. Curr. Eye Res..

[14] De Moraes C.G., Jasien J.V., Simon-Zoula S., Liebmann J.M., Ritch R. (2016). Visual Field Change and 24-Hour IOP-Related Profile with a Contact Lens Sensor in Treated Glaucoma Patients. Ophthalmology.

[15] Hollo G., Kothy P., Vargha P. (2014). Evaluation of continuous 24-hour intraocular pressure monitoring for assessment of prostaglandin-induced pressure reduction in glaucoma. J. Glaucoma.

[16] Mottet B., Aptel F., Romanet J.-P., Hubanova R., Pépin J.L., Chiquet C. (2013). 24-hour intraocular pressure rhythm in young healthy subjects evaluated with continuous monitoring using a contact lens sensor. JAMA Ophthalmol..

[17] Lorenz K., Korb C., Herzog N., Vetter J.M., Elflein H., Keilani M.M., Pfeiffer N. (2013). Tolerability of 24-hour intraocular pressure monitoring of a pressure-sensitive contact lens. J. Glaucoma.

[18] Dunbar G.E., Shen B.Y., Aref A.A. (2017). The Sensimed Triggerfish contact lens sensor: Efficacy, safety, and patient perspectives. Clin. Ophthalmol..

[19] Mansouri K. (2014). The road ahead to continuous 24-hour intraocular pressure monitoring in glaucoma. J. Ophthalmic Vis. Res..

[20] Mansouri K., Medeiros F.A., Tafreshi A., Weinreb R.N. (2012). Continuous 24-hour monitoring of intraocular pressure patterns with a contact lens sensor: Safety, tolerability, and reproducibility in patients with Glaucoma. Arch. Ophthalmol..

[21] Leonardi M., Leuenberger P., Bertrand D., Bertsch A., Renaud P. (2004). First steps toward noninvasive intraocular pressure monitoring with a sensing contact lens. Investig Ophthalmol. Vis. Sci..

[22] Tojo N., Otsuka M., Miyakoshi A., Fujita K., Hayashi A. (2014). Improvement of fluctuations of intraocular pressure after cataract surgery in primary angle closure glaucoma patients. Graefe’s Arch. Clin. Exp. Ophthalmol..

[23] Nganga Ngabou C.G.F., Makita C., Ndalla S.S., Nkokolo F., Madzou M. (2017). Intraocular pressure decrease after manual small incision cataract surgery. J. Fr. Ophtalmol..

[24] Aptel F., Musson C., Zhou T., Lesoin A., Chiquet C. (2017). 24-Hour Intraocular Pressure Rhythm in Patients with Untreated Primary Open Angle Glaucoma and Effects of Selective Laser Trabeculoplasty. J. Glaucoma.

[25] Muniesa M.J., Ezpeleta J., Benítez I. (2019). Fluctuations of the Intraocular Pressure in Medically Versus Surgically Treated Glaucoma Patients by a Contact Lens Sensor. Am. J. Ophthalmol..

[26] Mansouri K., Orguel S., Mermoud A., Haefliger I., Flammer J., Ravinet E., Shaarawy T. (2008). Quality of diurnal intraocular pressure control in primary open-angle patients treated with latanoprost compared with surgically treated glaucoma patients: A prospective trial. Br. J. Ophthalmol..

[27] Gillmann K., Bravetti G.E., Niegowski L.J., Mansouri K. (2019). Using sensors to estimate intraocular pressure: A review of intraocular pressure telemetry in clinical practice. Expert Rev. Ophthalmol..

[28] Tojo N., Hayashi A., Otsuka M., Miyakoshi A. (2016). Fluctuations of the Intraocular Pressure in Pseudoexfoliation Syndrome and Normal Eyes Measured by a Contact Lens Sensor. J. Glaucoma.

[29] Lozano D.C., Hartwick A.T., Twa M.D. (2015). Circadian rhythm of intraocular pressure in the adult rat. Chronobiol. Int..

[30] Dubey S., Mittal D., Mukherjee S., Bhoot M., Gupta Y.P. (2020). Relationship between nocturnal intraocular pressure-related peak recorded by contact lens sensor and disease progression in treated glaucomatous eyes. Indian J. Ophthalmol..

[31] Mansouri K., Gillmann K. (2020). Intereye Symmetry of 24-Hour Intraocular Pressure-related Patterns in Untreated Glaucoma Patients Using a Contact Lens Sensor. J. Glaucoma.

[32] Liu J.H., Mansouri K., Weinreb R.N. (2015). Estimation of 24-Hour Intraocular Pressure Peak Timing and Variation Using a Contact Lens Sensor. PLoS ONE.

[33] Leonardi M., Pitchon E.M., Bertsch A., Renaud P., Mermoud A. (2009). Wireless contact lens sensor for intraocular pressure monitoring: Assessment on enucleated pig eyes. Acta Ophthalmol..

[34] Currie B.D., Bagga H., Rademaker A.W., Tanna A.P. (2011). Effect of instrument orientation on the accuracy of intraocular pressure measurements in human cadaveric eyes: Manometric evaluation of the model 30 classic pneumatonometer and Tono-pen XL. J. Glaucoma.

